# Facilitating equitable access to hospice care in socially deprived areas: A mixed methods multiple case study

**DOI:** 10.1177/02692163221133977

**Published:** 2022-11-15

**Authors:** Maddy French, Thomas Keegan, Nancy Preston

**Affiliations:** 1 Division of Health Research, Lancaster University, UK; 2 Lancaster Medical School, Lancaster University, UK

**Keywords:** Socioeconomic factors, health disparities, palliative care, hospice and palliative care nursing

## Abstract

**Background:**

There is uncertainty about the factors influencing inequities in access to palliative care in socially deprived areas, including the role of service models and professional perceptions.

**Aim:**

To explore the relationship between social deprivation and access to hospice care, including factors influencing access and professional experiences of providing care.

**Design:**

A mixed-methods multiple case study approach was used. Hospice referrals data were analysed using generalised linear mixed models and other regression analyses. Qualitative interviews with healthcare professionals were analysed using thematic analysis. Findings from different areas (cases) were compared in a cross-case analysis.

**Setting:**

The study took place in North West England, using data from three hospices (8699 hospice patients) and interviews with 42 healthcare professionals.

**Results:**

Social deprivation was not statistically significantly, or consistently, associated with hospice referrals in the three cases (Case 1, Incidence Rate Ratio 1.04, *p* = 0.75; Case 2, Incidence Rate Ratio 1.09, *p* = 0.15, Case 3, Incidence Rate Ratio 0.88, *p* = 0.35). Hospice data and interviews suggest the model of hospice care, including working relationship with hospitals, and the local nature of social deprivation influenced access. Circumstances associated with social deprivation can conflict with professional expectations within palliative care.

**Conclusion:**

Hospice care in the UK can be organised in ways that facilitate referrals of patients from socially deprived areas, although uncertainty about what constitutes need limits conclusions about equity. Grounding professional narratives around expectations, responsibility, and choice in frameworks that recognise the sociostructural influences on end-of-life circumstances may help to foster more equitable palliative care.

**What is already known about this topic**

- Socioeconomic disadvantage is often associated with a lower likelihood of accessing specialist palliative care, with factors influencing access likely to be multi-faceted, relating to patients, services, and society.
- Working in healthcare in socially deprived areas can be rewarding but challenging, impacted by resource pressures, high patient need, and uncertainty about responding to social inequities.

**What this paper adds**

- This paper describes organisational, professional, and contextual factors that may facilitate hospice referrals from socially deprived areas.
- Circumstances associated with social deprivation can conflict with professional expectations within palliative care, triggering a range of responses from providers.

**Implications for practice, theory or policy**

- There is a need for greater clarity about what an equitable palliative care service may look like in practice, given local deprivation characteristics.
- Palliative care professionals may benefit from drawing on ethical frameworks that recognise socio-structural influences on inequities in end-of-life experiences.
- Close working between hospices and hospitals may facilitate referrals from socially deprived areas.

## Background

Facilitating access to palliative care is not the only way to overcome unfair differences in how people die but it can be a gateway to achieving better deaths. In many countries with well-developed palliative care services, access to that care varies by socioeconomic position.^1–4^ Widely used indicators of individual socioeconomic position include income, employment, or education, through which resources, assets and power are distributed across society.^5^ Better understanding of socioeconomic differences in access, and how they can be addressed, is one of many actions required to achieve policy goals of universal access to palliative care.^6–8^

One approach to addressing this need is to consider the socioeconomics of areas where people live. Area deprivation is a commonly used measure of area socioeconomics, often comparing population incomes, employment, educational qualifications and other measures using census data.^9–11^ While there is evidence that individual socioeconomic disadvantage is associated with lower likelihood of receiving specialist palliative care,^3^ evidence about area-level socioeconomic effects is arguably more uncertain and inconsistent, particularly in the UK.^12–14^

There is a growing body of literature exploring factors influencing access to palliative care for socioeconomically disadvantaged populations. These factors are likely to be multi-faceted and interacting, related to patients, families, professionals, services, and societal structures.^1,4,13,15^ Lifelong experiences of marginalisation may lead to mistrust among patients, exacerbated by poorly coordinated palliative care and stigmatisation by providers.^15^ Services can be geographically or culturally inaccessible, or organised in ways that are difficult to navigate and require high levels of health literacy and social support.^1,13,16^

Equitable access is not just about referrals. Inequities can feature in how healthcare professionals understand, identify, and respond to socioeconomic circumstances.^17^ When faced with challenges in socially deprived areas, including fewer resources and greater needs,^18,19^ healthcare professionals sometimes respond by focussing on individual behaviour change rather than structural causes of issues.^20,21^ Comparable evidence about palliative care professionals is more limited; a literature review highlighted the need for evidence about how palliative care professionals perceive the impact of social inequalities on ‘good dying’ and professional practice.^22^ Exploring this, alongside other access issues, will help identify what healthcare professionals need to support equitable high-quality palliative care for people experiencing social deprivation, identified as a priority research problem in this area.^4^

## Research aims

This study explores the relationship between social deprivation and accessing UK hospice care. UK hospices are charitably run organisations providing palliative care at home, in inpatient units, day centres, and other settings,^23^ predominantly in the last few months of life.^24^ Although palliative care services share a philosophy of holistic care, attending to physical, psychological, social, and spiritual needs, care delivery differs between settings.^25–27^ We explore these differences in relation to social deprivation and hospice access, focussing on:

1. Referrals, and factors influencing access, to hospice care in the UK.
2. Healthcare professionals’ experiences of providing generalist or specialist palliative care in the context of social deprivation.

### Study setting

This study setting is three hospice catchment areas in North West England, a region home to some of the most socially deprived areas in the UK. The setting includes small cities, towns, and rural areas, but no large cities. Many of the socially deprived areas are coastal, typically on the economic and geographic periphery of society with seasonal economies distinguishing this type of deprivation from that in urban areas.^28^ Most people (92%) in the North West are white^29^; the study areas had slightly higher proportions of white populations (Table 2).

**Table 1.** Case eligibility criteria.

	Case #1	Additional cases
Inclusion criteria	Contains areas that fall within the 10% most deprived and 10% least deprived nationally. Has a specific catchment area where services are delivered. Only one hospice service offering services within the majority of that catchment area.	Same inclusion criteria PLUS: Offers different types of services or delivers them in a different way.
Exclusion criteria	An unspecified catchment area or a significant overlap of populations shared with another hospice service provider.	Same exclusion criteria PLUS: No obvious differences in the type of services provided or how they are delivered.

## Methods

### A multiple case study approach

A mixed-methods multiple case study approach was used. Case study involves studying phenomena in-depth and in relation to the context of a particular case.^30,31^ Each of the three cases in this study was an example of ‘accessing hospice care via hospice referrals in socially deprived areas’ and included one hospice, selected because of differences in hospice service models. Table 2 outlines additional eligibility criteria. The hospice catchment area determined a case’s geographical boundaries. Data were collected and analysed within each case first, before comparing findings in a cross-case analysis.

**Table 2.** Area characteristics in Cases 1-3.

Variable	Case 1	Case 2	Case 3
Hospice services (included in study)			
Inpatient unit	Yes	Yes	No
Community-based service(s)	Yes	Yes	Yes
Hospital-based service	No	Yes	No
Hospice referrals			
Patients referred	2208	5626	865
Mean (SD) rate per 1000	11 (7.6)	22.9 (11.4)	7.8 (5.6)
Geography (%)			
Rural	50	9	61
Urban	50	91	39
Gender^a^ (%)			
Female	51	51	50
Male	49	49	50
Ethnicity^a^ (%)			
White	97	97	99
Other than white	3	3	1
Age^a^ (%)			
65 and over	24	24	22
Under 65	76	76	78
Distance^a^ (km)			
Min	0.5	0.35	0.3
Median	7.4	5.05	11.6
Max	51.5	15.3	52
Crude mortality^a^			
Rate per 1000	11.8	13.7	11.1

a Mean value for LSOAs in that case.

### Data collection

#### Routine and administrative data

Data on postcode, age, gender, diagnosis, date of referral, initial referrer, and initial service received were retrieved for individual patients referred to hospice care between September 2016 and September 2018 (Case 1), April 2016 and April 2019 (Case 2) and April 2016 and December 2019 (Case 3). Postcodes were replaced by Lower Layer Super Output Area (LSOA) codes, covering areas of approximately 1500 people, used to link to area social deprivation data from the Index of Multiple Deprivation.^32^ Data on area characteristics (deaths, population size, age structure, gender structure, ethnicity structure, rurality) were collected from Office for National Statistics datasets. Distance to hospice was straight-line distance from LSOA centroid to the main hospice building. Crude mortality rates were created using 2016 data on deaths and LSOA population.

Each LSOA has a national area social deprivation rank in the Index of Multiple Deprivation. LSOAs were ordered by deprivation rank and then re-ranked alongside other LSOAs in that hospice catchment area, creating regional deprivation ranks. Regional ranks are appropriate for case study methodology, where case analysis should be driven primarily by data from within each case,^30^ here focussing on within-case variation of deprivation. Distributions of national and regional deprivation ranks are provided in Supplemental Materials (Tables S1–S2).

#### Missing data

A patient’s initial referral was identified using their unique ID. This included some patients for whom the referrer had been coded as ‘hospice’. While suggestive of an internal referral, these were likely initial referrals that had been miscoded. Details of missing data are provided in Supplemental Materials (Table S3). No statistical method was used to address missing data; the implications were considered when interpreting findings.

#### Qualitative data

Referrers and hospice staff, with generalist and specialist palliative care experience, were recruited to take part in semi-structured qualitative interviews. All interviews were conducted by MF, a PhD student with no professional or personal connections to participants. Initial interviews were undertaken with senior hospice staff, with subsequent staff identified through snowball sampling. Referrers were contacted using details in the public domain or through snowball sampling. Participants received a participant information sheet and signed a consent form prior to participating. All interviews took place before February 2020.

A sequential mixed-methods design^33^ was used. In Cases 1 and 2, the quantitative results were analysed before commencing qualitative interviews. Some findings from quantitative results were explored in these interviews, for example patterns related to day hospice in Case 1. Specific needs of data providers in Case 3 meant qualitative interviews preceded, but did not influence the design of, quantitative data collection in that case.

### Data analysis

Each case was analysed individually (within-case analysis) before being compared (cross-case analysis).

#### Within-case analysis

##### Hospice referral rates (area analysis)

The first analysis examined the relationship between social deprivation and area hospice referrals (the outcome). Area geographical classification (rural/urban), gender (% of females), ethnicity (% of white people), age (% aged over 65), distance (km from LSOA to hospice), and crude mortality were included as covariates. Stepwise regression was used to select variables for multivariate Poisson mixed models, offset by LSOA population aged 20+, with LSOA added as an observational-level random effect to address overdispersion. Incidence rate ratios (IRRs) were generated with p values and 95% confidence intervals. A *p* value ⩽0.05 was selected a priori as a statistical significance threshold.

##### Hospice patient characteristics (individual analysis)

Separate multiple linear regression models were run to examine the relationship between social deprivation and routes into hospice care, focussing on (1) who referred patients and (2) initial service received. Age, gender, and diagnosis were included as covariates based on statistical significance in univariate linear regression analyses (*p* ⩽ 0.05). Deprivation rank was the outcome to allow comparison of the distribution of deprivation within each patient characteristic group category. For ease of interpretation, coefficients are reported as a percentage change.

##### Qualitative interviews

Qualitative interviews were analysed in each case using reflexive thematic analysis.^26,27^ Interview transcripts were read line-by-line and evidence about factors influencing access coded inductively. Codes were merged and relationships explored using concept mapping, creating themes offering feasible explanations for the statistical results or capturing wider reflections on providing care in socially deprived areas.

#### Cross-case analysis

The cross-case analysis began after all qualitative and quantitative analyses were complete for each case. The within-case findings provided the initial framework for the cross-case analysis, which drew on quantitative and qualitative data by comparing patterns in referrals and factors influencing access between cases. Using a pattern-matching approach,^30,31^ outcomes (referral rates, referrals to different hospice services, referrals of different diagnostic groups, etc.), explanatory factors, and context, were compared using matrices and concept mapping, and cross-case themes generated inductively.

### Ethical approval

Ethical approval for the study was provided in July 2018 by the NHS North West Greater Manchester West Research Ethics Committee (18/NW/0460). Participating hospices provided organisational approval.

## Results

Data from 8699 hospice patients and 42 healthcare professionals contributed to findings. Area and hospice patient characteristics are provided in Tables 1 and 3. Details of interview participants are provided in Table 5.

**Table 3.** Hospice patient characteristics in Cases 1–3.

Variable	Category	Case 1		Case 2		Case 3	
		*N*	%	*N*	%	*N*	%
Deprivation	Most deprived – 1	540	24	975	17	176	20
	2	395	18	1076	19	171	20
	3	393	18	1357	24	128	15
	4	382	13	1224	22	179	21
	Least deprived – 5	498	23	994	18	211	24
Initial service received	Day hospice	419	19	252	4.48	-	-
	Hospice at home	760	34	1167	21	865	100
	Inpatient hospice	563	26	52	0.9	-	-
	Hospital	-	-	2508	45	-	-
	Community	-	-	1604	29	-	-
	Outpatient consultant	-	-	43	0.8	-	-
	Missing	466	21	-	-	-	-
Initial referrer	GP surgery^a^	509	23	748	13	-	-
	Clinical nurse specialists in palliative care	234	11	-	-	266	31
	Community (district nurses; community matrons etc.)	293	13	80	14	141	16
	Hospital	434	20	3491	62	157	18
	Other	212	10	6	0.1	63	7
	Other HCP	–	–	–	–	195	23
	Missing *(no referrer specified)*	60	3	–	–	1	0.1
	Missing *(referrer specified as hospice)*	466	21	143	3	3	0.4
Diagnosis	Cancer	1393	63	2829	50	602	70
	Non-cancer	662	30	1757	31	263	30
	Missing	153	7	1040	18	–	–
Gender	Female	1134	51	2965	53	443	51
	Male	1074	49	2661	47	422	49
Age	Mean	75	–	77	–	78	–
	Min	19	–	17	–	21	–
	Max	100	–	107	–	107	–

a Fewer than 2% of referrals in Case 3 came from GP surgeries; these were included in the ‘community’ category.

Estimated incidence rate ratios (IRRs) (Table 4) compare the 20% most deprived areas in each case to all other areas, holding other covariates constant. In Case 1, the most deprived areas are estimated to have a referral rate 1.04 times as great as other areas, in Case 2 1.09 as great, and in Case 3 0.88 as great, although no estimates reached statistical significance (Case 1, *p* = 0.75; Case 2, *p* = 0.15; Case 3, *p* = 0.35; Table 4).

**Table 4.** Changes to hospice referral rates in the 20% most deprived areas compared to all other areas in each case.^a^

Social deprivation variable	Incidence Rate Ratios (IRR)	p Value	95% CI
**Case 1 (adjusted for crude mortality, population ethnicity, population gender, distance to hospice)**			
Deprivation quintiles 2–5	Reference factor		
Deprivation quintile 1 (20% most deprived)	1.04	0.75	0.92–1.33
**Case 2 (adjusted for crude mortality, population age, population gender, urbanity, distance to hospice)**			
Deprivation quintiles 2–5	Reference factor		
Deprivation quintile 1 (20% most deprived)	1.09	0.15	0.97–1.24
**Case 3 (adjusted for crude mortality and distance to hospice)**			
Deprivation quintiles 2–5	Reference factor		
Deprivation quintile 1 (20% most deprived)	0.88	0.35	0.66–1.16

a Variation of observation-level random effect for LSOA reported in Table S6.

**Table 5.** Distribution of interview participants across cases.

	Hospice staff	Referrers (community)	Referrers (hospital)	Total
Case 1	4	7	0	11
Case 2	6	4	4	14
Case 3	2	13	2	17
Total	12	24	6	42

Table 6 reports the estimated percentage change in patients’ area social deprivation rank when comparing routes into hospice care. A higher rank equates to living in a less deprived area. Therefore, a percentage increase indicates that route into hospice care was associated with living in a less deprived area. In Case 1, compared to patients initially referred to day hospice, patients initially referred to the inpatient unit, came from areas that were on average 4.43% less deprived; those initially referred to hospice at home came from areas 3.8% less deprived. This is a small but statistically significant change in deprivation rank. While it is difficult to discern the absolute changes in deprivation experiences between these groups, as with any use of deprivation ranks we can conclude that on average patients initially referred to day hospice in Case 1 lived in areas of greater deprivation across income, employment, and other domains. Hospice patients initially referred from hospital settings in general lived in more socially deprived areas that those referred by GP surgeries in Case 2 and by community services in Case 3 (Table 6).

**Table 6.** Percentage change in area social deprivation rank associated with change in patient characteristic calculated from multivariate linear regression (adjusted for age). An increase indicates an association with patients in less deprived areas compared to the reference; a decrease indicates an association with patients from the most deprived areas.

Case			*p* Value
Case 1			
Initial service received	Day hospice	(reference)	
	Inpatient hospice	4.43	<0.05
	Hospice at home	3.80	<0.05
Case 2			
Initially referred by	Hospital	(reference)	
	GP surgery	2.89	<0.01
	Community	−4.87	0.11
	Out of hours	−0.43	0.65
	Other	20	<0.05
Case 3			
Initially referred by	Hospital	(reference)	
	Clinical nurse specialists	8.29	<0.01
	District nurses	7.65	<0.05
	Informal	9.90	0.06
	Other (non-HCP)^a^	8.23	0.07
	Other (HCP)^a^	4.16	0.2

a HCP = Healthcare Professional.

### Cross-case themes

#### Tensions arising from local and lifelong experiences of deprivation

Participants often emphasised different characteristics of social deprivation, depending on the case in which they worked. In the first, participants highlighted the prevalence of unmanaged mental health illness. In the second, they emphasised transient populations, social isolation, and substance use; in the third, large and local families. Consequently, the impact on palliative and end-of-life care was characterised differently:

they often have very good family support because these areas are often quite, sort of, you know, they have the traditional family model still. . . I don’t see social deprivation has a huge impact on the end-of-life care. (Ian, Referrer GP, Case 3)

Drug misuse is very high. . .and so when we got a patient who is on a huge dose of morphine, or diamorphine, in a syringe driver. . . [it is complex to] keep them safe. . . (Kate, Hospice Senior Staff, Case 2)

When reflecting on these circumstances, participants conveyed a tension between their expectations of their role and the reality of caring for people in socially deprived circumstances. This included having insufficient psychological training to support people with serious mental health needs, working in poor quality and insecure housing, or struggling to overcome long held mistrust of formal services:. . .for quite a while she was very, very standoffish. She really didn’t want to know. . . But it was about “well you know how you gonna help me? Nobody helped me in the past”. Quite flippant. (Val, Hospice Senior Nurse, Case 2)

Accounts of mistrust or anger at services co-occurred alongside descriptions of low expectations of care or people being ‘grateful for whatever they get’ in more deprived areas (Carol, Hospice Senior Nurse, Case 2). While perceived differently, gratefulness and anger both related to local, long-term issues accessing services. These accounts show how experiences accessing care at the end-of-life cannot be divorced from the historical and local experience of social deprivation. For some participants, the impact of these lifelong experiences for patients created a tension between the ideal and the reality of providing palliative care in socially deprived areas.

#### Connecting with patients at moments of vulnerability

In each case, there was a hospice referral route associated with living in a more socially deprived area. In the first, this was being initially referred to day hospice (Table 6). In the other cases, referrals from hospitals were important. Some referrers suggested these routes corresponded to patients in these areas seeking help following crises or heightened emotions:

. . .especially if they are an inpatient in hospital, they are often. . .in more vulnerable states. They’re often perhaps more amenable to having that conversation. (Jane, Referrer GP, Case 2).

. . .there is a lot of anxiety which makes their breathing worse anyway which makes them see you more. . . So, as a GP, it kind of boils down to well how can we minimise how much you come see me. The day hospice is very good, we’ve had some good experience, let’s send you for some of that. (Jonathan, Referrer GP, Case 1)

Seeking healthcare at moments of vulnerability coincided with flexibility in the hospices’ remits. This included adapting day hospice to a wider range of patients, responding to referrers’ needs, acknowledging some patients will only be reached in hospital, and accepting ‘generalist’ referrals. While engaging with community referrers was important, collaboration between hospices and hospitals appeared to facilitate referrals from socially deprived areas.

#### Negotiating and managing professional input

Participants reflected on the amount and type of care provided in socially deprived areas. The sub-theme ‘Bridging the gap between patients and services’ describes how participants negotiated care; ‘Relating needs to resources’ describes how this work was bounded by perceptions of capacity.

##### Bridging the gap between patients and services

Participants described bridging gaps between services and people in socially deprived areas. This involved negotiating *with* patients to align expectations of services with those of professionals, including by exchanging skills and knowledge (e.g. resilience, self-efficacy, system knowledge) and material assets (financial grants, buying household items):

I see ‘well lass what can I do? You know, there’s nowt I can do about it so let’s just get on’. . .. I do a lot of work about what we can do. . .how are we going to manage things and we negotiate a way forward (Sheila, Referrer Specialist Nurse, Case 3)

Participants also negotiated *for* patients, using knowledge and power to facilitate access. This required relationship management across diverse public services, enabling participants to ‘pull strings’ (Adi, Referrer Specialist Nurse, Case 1) for patients impacted by socioeconomic circumstances. Hospices with internal community clinical nurse specialist teams appeared more involved in this.

##### Relating needs to resources

Some participants working in socially deprived areas discussed needing more time to build relationships or organise facilities for home care, sometimes leading to lengthy, multiple visits. Participants sometimes perceived family input to lessen this need, although the psychosocial needs of families impacted by deprivation could result in substantial service input.

Reflections on service input coincided with observations about capacity. Opinions differed on a hospice’s capacity to respond to a local area’s broad needs, whether by filling gaps in other sectors or expanding services to new populations:

. . .the GPs who are [in socially deprived areas], it’s just, I don’t know how they are carrying on. . .so I have to go to them sort of saying how can I help you, how can I reduce your workload. . . (Becky, Hospice Senior Doctor, Case 2)

. . .it’s a great thing but you are spreading yourself thinner and thinner . . . We’ve opened up our doors now . . . and you know granted its the right thing and we want to be able to support people as best we can but its definitely. . .a change. (Val, Hospice Senior Nurse, Case 2)

In Case 1, there was a sense that limited referrer capacity in socially deprived areas increased referrals, whereas other primary care services had ‘the time to do it’ themselves (Hannah, Hospice Senior Staff, Case 1). However, uncertainty about the appropriateness of this meant participants were not sure whether this was equitable or inequitable.

#### Distress, risk, and choice in professional responses

Participants described a range of responses to professionally challenging situations encountered in the context of social deprivation, ranging from resilience and flexibility to distress and the withdrawal of care:

And the other team who have the vast majority of the deprived areas with issues. That team just get on with it, they never get distressed by anything . . . But the other team, because it’s not very often, you’ve got an awful lot supporting them [to do] alongside trying to sort and help the situation get better. And the [hospice] team, helping them because they are finding it distressing because it’s not a perfect death. Yet [the family’s] interpretation of that was [they were] supported absolutely fantastically. (Adi, Referrer Specialist Nurse, Case 1)

Others also suggested that the extent to which professionals encountered challenges influenced their response, including how risk-averse teams were. For example, Arlene, working in socially deprived areas where substance dependency was perceived as rare, responded differently to Kate, for whom this circumstance was more common:

I have a lady who has high alcohol intake who lives in quite dire circumstances. . .She’s quite a vulnerable person because she tends to have different individuals in and out of her flat on a daily basis. So obviously we’ve highlighted to her that this will affect her end-of-life care because, one, I can’t prescribe any opioids to put in that environment. . . worst case scenario is that this lady could end up being deprived of end-of-life input because she’s deemed to have capacity and she’s putting herself in that position. (Arlene, Referrer Specialist Nurse, Case 3)

. . .we certainly had a couple of patients, who were part of an IV drug misuse er, er, gang I suppose. . . how safe is it for us to send that patient home, which is where they wanted to go, but with a whole stock of controlled drugs which their colleagues would like to sell. So, the way we solved that was he had a very limited supply and we monitored it as closely as we could. Sadly, they still did misuse these drugs but it was his choice, he wanted to go back to that. (Kate, Hospice Senior Doctor, Case 2)

The quotes from Kate and Arlene also exemplify how patient choice and agency featured in participants’ interpretations of outcomes in socially deprived areas. Participants sometimes drew on these concepts to resolve tensions between professional aspirations for palliative care and what was considered achievable, given the disadvantaged circumstances of some patients’ lives. Focussing on whether a patient or family was seen to be making ‘genuine’ choices or had capacity made it easier to accept decisions considered unwise by some professionals. As well as featuring in professional responses to home environments, choice was also used to justify patients dying alone or resisting input from services. Some participants acknowledged the rationale behind these decisions, given a patient’s lifelong experiences:

Sometimes they do just disappear off and we can’t see them and you know you just have to accept that they will just say I’ve had enough. Why would you trust someone? You’ve been on the streets since 12, you know . . . they’re not going to just [think] ‘oh you’re the nice palliative care doctors’ (Becky, Hospice Senior Doctor, Case 2)

Agency also featured in praise for patients and families seen to manage care independently in the context of social deprivation. People seen as not making a fuss or not needing ‘to have carers or anything because they took it in turns as a family’ (Rachel, District Nurse, Case 2) were perceived positively, indicating the relevance of agency when interpreting both positive actions and those seen to misalign with palliative care aspirations.

## Discussion

### Main findings

The aim of this study was to explore the relationship between social deprivation and access to hospice care. We did not find consistent or certain evidence of differences in referrals between more and less socially deprived areas. The quantitative and qualitative data suggested that aspects to do with service organisation and delivery (the model of care), including relationship with hospitals and service flexibility, influenced how hospices connected with people from socially deprived areas. Professional expectations about the role of palliative care, such as psychologically supporting people or caring for them safely at home, were challenged by situations encountered in some socially deprived areas, such as high levels of unmet mental health need or substance dependency. The understanding and provision of equitable care was underpinned by the local nature of social deprivation and professionals’ perception of capacity and responsibility.

### Strengths and limitations

Contextualising the referrals analysis by qualitatively exploring factors influencing access in each case allowed us to explore local factors and commonalities between areas. However, difficulties measuring population need for hospice care substantially limited conclusions about the equity of referrals. Knowing the number of patients not referred to palliative care, but who could have benefitted, would enable more precise and robust estimates. Mortality rates, used in this study to attempt to adjust for population need, are unlikely to capture relevant complex needs,^34^ and area deprivation did not adequately capture the diverse local experiences of area socioeconomics that influenced access in this study.

Lacking input from patients and families, some important factors may have been missed, including stigmatising attitudes within healthcare settings.^15^ Focussing on access to formal services also meant that informal community support was not explored.^35^ Given no large cities were included in this study, the findings may not easily transfer to metropolitan urban areas of deprivation. However, the transferability of findings to smaller cities, towns, and coastal areas is also a strength of our study, as there is a need to better understand patterns of deprivation and inequity in these areas.^28^

### Implications for practice and research

Efforts to improve access to hospice care in socially deprived areas would benefit from adapting to local assets and challenges, understanding the role of both hospitals and community settings, and being clear about what constitutes an equitable service. That professional expectations of palliative care may be challenged when working in socially deprived areas calls for reflection in practice about how professionals respond to these challenges. This could be done through introducing structural competency training^36^ and by adopting alternative ethical frameworks for explaining and understanding unfair social differences in palliative care.^37,38^ Further discussion of these implications is included below.

The findings call for research on the role of hospital-hospice partnerships in helping to address socioeconomic differences in hospice referrals. Ensuring the experiences of patients and families with lived experience of social deprivation are included in future research is paramount, particularly for exploring issues around need, choices, and preferences.

### Referrals and factors influencing access

Given that a meta-analysis indicated individual socioeconomic disadvantage is associated with lower odds of receiving specialist palliative care, our study findings may not reflect experiences in other areas.^3^ Indeed, a study in a nearby city found evidence of lower hospice at home referral rates in socioeconomically disadvantaged areas.^12^ However, there is some uncertainty in the UK about the consistency of the relationship between area deprivation and access to palliative care.^13^ The findings from our study suggest that this uncertainty may relate to the diverse characteristics of social deprivation and models of care.

In this study, when it was common for families to stay in a local area, service providers sometimes perceived less need for their input. However, where this combined with perceived poor provision of local mental health services, some providers suggested psychosocial family needs at the end-of-life increased. While some causes of inequities in end-of-life circumstances may lie beyond the influence of local communities, these findings suggest that efforts to foster more equitable palliative care provision would benefit from identifying and responding to local assets and challenges in socially disadvantaged areas. These could draw on place-based approaches used in other work on community responses to social determinants of health.^38,39^ Importantly, however, the characteristics that study participants related to social deprivation, such as family size, family presence, mental health, and substance use, reflected their perceptions of particular areas. These may differ to the perspectives of other people living and working in those or other similar areas, differences that should be acknowledged when assessing local assets and challenges.

Hospitals may play an important role in initially connecting people in socially deprived areas with hospice care, and potentially other specialist palliative care services. This adds to evidence challenging the ‘problematisation’ of hospitals within palliative care policy, where hospitals tend to be discussed in relation to unmet place of death preferences, unnecessary hospital admissions, or poor quality care.^40^ Greater use of hospitals at the end-of-life among people experiencing socioeconomic disadvantage is well documented, thought to partly relate to poorer health.^3,41^ Our findings highlight how close working between hospices and hospitals, and potentially greater patient vulnerability in that setting, may facilitate referrals from socially deprived areas.

While efforts to address inequities in access to palliative care should focus on community settings, it is unlikely this can overcome all the reasons why people in socially deprived areas are more likely to end up in hospital. Hospice and other specialist palliative care organisations could look to foster closer integration with hospitals, as well as community settings, as a way of initially connecting with patients in more socially deprived areas. Policy aimed at addressing inequities in access should acknowledge the distinct but related roles of hospital and community services in this endeavour.

### Responsibility for responding to socioeconomic inequities at the end-of-life

Uncertainty about responding to socioeconomic disadvantage in this study features in other research with healthcare professionals about responsibility to act upon inequities, and what form that should take.^18,42^ Others working in socially deprived areas also speak about a tension between the ‘hidden work’ they do for patients failed by the system and a desire to define and protect their role.^43^ The importance of attending to a person’s holistic needs in palliative care may add to uncertainty about boundaries of care in the context of social deprivation, where needs may be beyond the existing skillset or historical remit of palliative care. With the potential for this to shift expectations of palliative care and what it means to die well, attention should be paid to the intuitions and assumptions influencing professional attitudes towards socioeconomic inequities.

Some participants drew on patient choice narratives to help rationalise professionally challenging situations encountered in socially deprived areas. It is not uncommon for healthcare professionals to draw on such narratives when faced with the effects of social inequities, possibly reflecting a clinical focus on diagnosing and treating individuals.^20^ This has implications for access to palliative care because it risks placing the burden of responsibility for addressing barriers to access associated with socioeconomic disadvantage on the shoulders of patients and families. Other equity studies in palliative care have highlighted how discourses on autonomy, agency, and choice reproduce inequities, for example by suggesting that female caregivers are responsible for ensuring they can provide voluntary care.^44 ^45^^ When seeking to address inequities in access to palliative care, the language used by professionals should acknowledge the socio-structural influences on the agency and choices people have at the end-of-life. In this way, challenges could be reframed from a tension between professional expectations and patient choice, as implied by some participants in this study, to one between palliative care goals and the socio-political structures that lead to inequities.

### Implications for theory and its use in practice

The study findings support the argument that access to healthcare plays out in interactions between patients and healthcare professionals, who jointly negotiate a person’s ‘candidacy’ for care.^17^ However, ‘access’ is also shaped by forces external to those interactions. For example, how services are organised can determine the amount of work required from people to ‘fit’ into those services.^17,46^ Practical efforts to improve equitable access to palliative care should draw on theories that recognise the structural forces perpetuating inequities and the power that different groups have to act on those forces.^36,38,47–49^ This could help delineate the responsibilities of professionals, patients, communities and political institutions in efforts to achieve more equitable access to palliative care.

### Conclusion

Hospice care in the UK can be organised in ways that facilitate referrals from socially deprived areas, although uncertainty about need limits conclusions about equity. Referrals to hospice were influenced by the nature of social deprivation particular to each case. How hospices had chosen to organise their services also influenced how and where they connected with people from the most deprived areas. Greater reflection and discussion about responsibility and choice may help support professionals responding to socioeconomic inequities at the end-of-life.

## Supplemental Material

sj-pdf-1-pmj-10.1177_02692163221133977 – Supplemental material for Facilitating equitable access to hospice care in socially deprived areas: A mixed methods multiple case study Click here for additional data file. Supplemental material, sj-pdf-1-pmj-10.1177_02692163221133977 for Facilitating equitable access to hospice care in socially deprived areas: A mixed methods multiple case study by Maddy French, Thomas Keegan and Nancy Preston in Palliative Medicine
