# Supplementary material for: Facilitating equitable access to hospice care in socially deprived areas: A mixed methods multiple case study
Source: Palliat Med. 2022 Nov 15;37(4):508–19. doi: 10.1177/02692163221133977 (PMC10074748; doi:10.1177/02692163221133977)
Supplement: sj-pdf-1-pmj-10.1177_02692163221133977 – Supplemental material for Facilitating equitable access to hospice care in socially deprived areas: A mixed methods multiple case study [file sj-pdf-1-pmj-10.1177_02692163221133977.pdf]

Facilitating equitable access to hospice care in socially deprived areas: a mixed methods multiple case study

Supplementary Materials

Appendix 1

Table S1: Distribution of national area deprivation quintiles in each case

	Case 1		Case 2		Case 3	
	N	%	N	%	N	%
Quintile 1 (most deprived 20%)	21	13	61	30	23	25
Quintile 2	20	13	43	21	26	28
Quintile 3	34	22	45	22	21	23
Quintile 4	55	35	35	17	11	12
Quintile 5 (least deprived 20%)	27	17	22	11	11	12

Table S2: Distribution of regional area deprivation quintile in each case

	Case 1		Case 2		Case 3	
	N	%	N	%	N	%
Most deprived - 1	32	20	42	20	19	20
2	31	20	41	20	18	20
3	31	20	41	20	18	20
4	31	20	41	20	18	20
Least deprived -5	32	20	41	20	19	20

Appendix 2

Table S3: Missing data by patient and provider characteristics in Case 1

	Category	N	%
Service	Day Hospice	419	19
	Hospice at Home	760	34
	Inpatient	563	25
	**Missing**	**466**	**21**
Referrer	Clinical nurse specialist	234	11
	District Nurse	293	13
	GP Surgery	509	23
	Hospice (internal)	466	21
	Hospital	434	20
	Other	212	10
	**Missing**	**60**	**3**
Diagnosis	Cancer	1393	63
	Non-cancer	662	30
	**Missing**	**153**	**7**

Table S4: Missing data by patient and provider characteristics in Case 2

	Category	N	%
Diagnosis	Cancer	2829	50
	Non-cancer	1757	31
	**Missing**	**1040**	**18**

Table S5: Missing data by patient and provider characteristics in Case 3

	Category	N	%
Referrer	Clinical Nurse Specialists	266	31
	District Nurse	141	16
	Hospital	157	18
	Informal	39	5
	Hospice (internal)	3	0.3
	Other (non-HCP)[1]	63	7
	Other HCP[1]	195	23
	**Missing**	**1**	**0.1**

[1] HCP=Healthcare Professional

Appendix 3

Table S6: Standard deviation of observation-level random effect for LSOA (OLRE-SD) in each case analysis

Case	OLRE-SD[1]	Number of LSOAs
Case 1	1.64	157
Case 2	1.23	206
Case 3	1.50	92

[1] Exponentiated OLRE-SD generated by Poisson mixed models
